# Immunization with Human Cytomegalovirus Core Fusion Machinery and Accessory Envelope Proteins Elicit Strong Synergistic Neutralizing Activities

**DOI:** 10.3390/vaccines8020179

**Published:** 2020-04-13

**Authors:** Xinle Cui, Zhouhong Cao, Shuishu Wang, Stuart P. Adler, Michael A. McVoy, Clifford M. Snapper

**Affiliations:** 1 The Institute for Vaccine Research and Department of Pathology, Uniformed Services University of the Health Sciences, Bethesda, MD 20814, USA; 2 Vaccine Research Center, National Institute of Allergy and Infectious Diseases, National Institutes of Health, Bethesda, MD 20892, USA; 3 CMV Research Foundation, Richmond, VA 23229, USA; 4 Department of Pediatrics, Virginia Commonwealth University, Richmond, VA 23298, USA

**Keywords:** human cytomegalovirus, solid organ and hematopoietic stem cell transplantation, congenital infection, HCMV vaccine development, core fusion machinery, envelope glycoprotein, gB, gH/gL, UL128/UL130/UL131A, neutralizing antibody

## Abstract

Human cytomegalovirus (HCMV) core fusion machinery proteins gB and gH/gL, and accessory proteins UL128/UL130/UL131A, are the key envelope proteins that mediate HCMV entry into and infection of host cells. To determine whether these HCMV envelope proteins could elicit neutralizing activities synergistically, we immunized rabbits with individual or various combinations of these proteins adsorbed to aluminum hydroxide mixed with CpG-ODN. We then analyzed serum neutralizing activities with multiple HCMV laboratory strains and clinical isolates. HCMV trimeric gB and gH/gL elicited high and moderate titers of HCMV neutralizing activity, respectively. HCMV gB in combination with gH/gL elicited up to 17-fold higher HCMV neutralizing activities compared to the sum of neutralizing activity elicited by the individual proteins analyzed with both fibroblasts and epithelial cells. HCMV gB+gH/gL+UL128/UL130/UL131A in combination increased the neutralizing activity up to 32-fold compared to the sum of neutralizing activities elicited by the individual proteins analyzed with epithelial cells. Adding UL128/UL130/UL131A to gB and gH/gL combination did not increase further the HCMV neutralizing activity analyzed with fibroblasts. These data suggest that the combination of HCMV core fusion machinery envelope proteins gB+gH/gL or the combination of gB and pentameric complex could be ideal vaccine candidates that would induce optimal immune responses against HCMV infection.

## 1. Introduction

Human cytomegalovirus (HCMV) belongs to the human herpesvirus (HHV) family, and HCMV is also known as HHV5. HCMV is a double-stranded enveloped DNA virus and causes infection in more than 50% of the world population [1,2]. HCMV infection of immunocompetent individuals usually causes no symptoms, but HCMV infection of immunocompromised individuals such as transplant recipients and patients with HIV/AIDS causes significant morbidity and mortality [1,2,3,4,5]. Congenital HCMV infection of fetuses in the uterine causes serious health problems, and it is the leading cause of sensorineural hearing loss in childhood [1,2,6,7,8]. Congenital HCMV infection also causes microcephaly, intracranial calcifications, seizures, cerebral palsy, hepatitis and chorioretinitis resulting in vision loss [1,2,6,7,8]. HCMV infection in transplantation recipients not only causes hepatitis and pneumonitis, HCMV viremia can also significantly increase the chance of graft rejection and graft failure in solid organ transplant patients, as well as graft-versus-host disease in hematopoietic stem cell transplant patients [9,10,11,12]. Despite the fact that there have been many preventive procedures and treatments developed for controlling HCMV viremia in solid organ and hematopoietic stem cell transplant recipients such as active monitoring and management with novel and highly efficient antiviral drugs, HCMV infection is still one of the most common complications that negatively affects patient survival [13,14,15,16,17]. 

HCMV is spread horizontally via saliva, urine and possibly sexual contact, and across the placenta to the fetus [18,19]. Though HCMV infects a wide variety of cell types including fibroblasts, epithelial cells, endothelial cells, neurons, hepatocytes, monocytes and macrophages, HCMV infection of fibroblasts and epithelial cells represent the two distinct pathways of HCMV infection [20,21,22,23]. Like other members of the HHV family, HCMV enters host cells by fusing its envelope with either the plasma membrane or endosomal membrane, using multiple conserved envelope proteins, especially gB, gH and gL, which constitute the core fusion machinery of HHV [20,21,22,23,24,25,26,27,28]. For HCMV infection of and entry into fibroblasts, besides the core fusion machinery envelope proteins of HCMV gB, gH and gL, an additional envelope protein gO is required, where a protein complex formed by gH, gL and gO mediates binding to fibroblasts [21,22,23]. PDGF-α on the cell surface of fibroblasts serves as the receptor for the gH/gL/gO protein complex. The binding of gH/gL/gO protein complex to PDGF-α activates the gB protein, and triggers HCMV virion envelope and cell membrane fusion through macropinocytosis [24,25,26,29,30]. HCMV infection of epithelial cells requires accessory proteins UL128, UL130 and UL131A in addition to gB, gH and gL, the core fusion machinery envelope proteins [20,23,30,31,32]. There are also reports that gO is also required [20,23]. Accessory proteins UL128, UL130 and UL131A form a protein complex UL128/UL130/UL131A, and subsequently interact with gH and gL to form a pentameric complex gH/gL/UL128/UL130/UL131A [20,23,30,31]. Recent studies suggest that there are several receptors for the pentameric complex on epithelial cells, such as neuropilin-2, CD147, CD46 and OR14I1, and neuropilin-2 is the one that has been mostly studied [30,33]. The pentameric complex binds to neuropilin-2 on the epithelial cell membrane, triggering endocytosis, activating gB, followed by the fusion of the HCMV virion envelope with the epithelial cell endosomal membrane [30,33]. 

The development of a prophylactic vaccine for prevention of HCMV infection is a public health priority. A variety of experimental vaccine approaches have been evaluated over the past 50 years. Although live attenuated, subunit, DNA/RNA and viral vectored HCMV vaccines have each demonstrated promising results, none of them meet the requirements of licensing [34,35,36,37]. An HCMV recombinant gB vaccine adjuvanted with MF59 has advanced the furthest in clinical trials [38,39]. For the past several years, the HCMV recombinant monomeric gB/MF59 vaccine has completed three phase II clinical trials and demonstrated ~50% efficacy in prevention of HCMV infection [19,40,41,42,43]. The recombinant gB protein used in these clinical trials was expressed as a truncated polypeptide with mutations to facilitate protein secretion, but the gB did not recapitulate native HCMV gB conformation, which is a trimeric glycoprotein (44). Since the native conformation of the HCMV gB is a trimer, recombinant HCMV gB proteins that allow expression of quaternary conformational epitopes could potentially elicit broader, more robust and more protective vaccine responses [44,45]. 

HCMV gH/gL are essential components of the pentameric complex, and gH/gL alone can elicit potent neutralizing HCMV activity. Though the pentameric complex could induce significantly higher HCMV neutralizing activity than gH/gL, the antibodies elicited are mainly against the conformational epitopes formed by all five proteins and demonstrated mainly protection for epithelial and endothelial cells [38,46,47,48,49]. In contrast, the antibodies elicited by gH/gL have shown protection for fibroblasts as well as epithelial and endothelial cells [47,50,51]. HCMV gH/gL expressed in an alphavirus replicon particle (VRP) vaccine was reported to elicit potent and broadly cross-reactive HCMV neutralizing activities [50]. A human monoclonal anti-gH/gL antibody MSL-109 was demonstrated to protect fibroblasts from infection by multiple clinical and laboratory HCMV strains and was able to recognize HCMV gH protein complexes [51]. The pentameric complex has been extensively studied as an HCMV vaccine candidate in recent years due to its ability to elicit superior HCMV neutralizing activity for protection of epithelial and endothelial cells. Analysis of HCMV hyperimmune globulin demonstrated that the majority of the HCMV neutralizing activity preventing epithelial cell entry was against the pentameric complex, and the neutralizing activity against HCMV entry of epithelial cells decreased 85% after depletion with pentameric complex [52]. This is consistent with the report that the neutralizing activity preventing HCMV infection of epithelial cells from the sera of 365 HCMV seropositive women was 8- to 15-fold higher than that of fibroblast cells [53]. Further, recombinant pentameric complex elicited 100- to 1000-fold higher titers of HCMV neutralizing activity after immunization in mice as compared to that found in human subjects recovered from primary HCMV infection [46]. 

We previously reported the production of an HCMV trimeric gB [54]. Compared to a monomeric gB similar in structure to the HCMV gB that was used in clinical trials, HCMV trimeric gB induced significantly higher serum titers of gB-specific IgG after immunization in mice and elicited markedly higher titers of both complement-independent and complement-dependent HCMV neutralizing activities [54]. More importantly, the markedly higher titers of HCMV neutralizing activities elicited by trimeric gB demonstrated cross-strain protection against an HCMV AD169 strain expressing pentameric complex (AD169^wt131^) as well as several clinical HCMV strains [54]. 

To determine if HCMV core fusion machinery envelope proteins gH, gL, gB and accessory proteins UL128, UL130 and UL131A could elicit neutralizing antibodies synergistically, we produced recombinant HCMV gH/gL and UL128/UL130/UL131A proteins using a similar approach as that of HCMV trimeric gB. We then performed immunizations in rabbits with individual and various combinations of gH/gL, gB and/or UL128/UL130/UL131A proteins. The results of these studies will have important implications for future HCMV vaccine design.

## 2. Materials and Methods 

### 2.1. Reagents, HCMV Strains, and Cell Lines 

The DG44 cell line of Chinese hamster ovary (CHO) cells was cultured in CD DG44 serum-free medium supplemented with 8 mM L-glutamine (Thermo Fisher Scientific, Waltham, MA). ARPE-19 and MRC-5 cell lines were purchased from American Type Culture Collection (ATCC) and maintained using DMEM/F-12K or EMEM medium supplemented with 10% fetal bovine serum, respectively. HCMV strain AD169 was purchased from ATCC and propagated in MRC-5 cells. HCMV strain AD169^wt131^, a gift from Drs. Xiao Wang and Haruhiko Murata (United States Food and Drug Administration), was propagated in ARPE-19 cells [55]. HCMV low passage clinical isolates 38532 and 39621, and HCMV strains TS15-rN and UxcA66 were provided by Dr. Michael McVoy (Virginia Commonwealth University) [56]. HCMV clinical isolates 38532 and 39621 were propagated in MRC-5 cells, and HCMV strains TS15-rN and UxcA66 were propagated in ARPE-19 cells. The anti-gH antibody (0861, Santa Cruz Biotechnology, Dallas, TX, USA) was a mouse IgG1 monoclonal antibody, and a polyclonal rabbit anti-UL128 antibody was provided by Dr. Michael McVoy [56]. Goat anti-rabbit and goat anti-mouse secondary antibodies labeled with horseradish peroxidase were purchased from Thermo Fisher Scientific (Waltham, MA). Free-style Max reagent, pOptiVEC™-TOPO™ TA Cloning™ Kit and HisPur cobalt purification kit were also purchased from Thermo Fisher Scientific (Waltham, MA, USA). Superdex 200 column was purchased from GE Lifesciences (Pittsburgh, PA, USA). The polyclonal goat anti-rabbit IgG labeled with alkaline phosphatase was purchased from Southern Biotechnology (Birmingham, AL, USA), and the alkaline phosphatase substrate, disodium p-nitrophenyl phosphate, was from Sigma-Aldrich (St. Louis, MO, USA). The anti-IE1 monoclonal antibody MAB810 was purchased from Millipore (Burlington, MA, USA), biotin labeled goat anti-mouse secondary antibody from Jackson ImmunoResearch Labs (West Grove, PA, USA), and VECTASTAIN ABC reagent from Vector Labs (Burlingame, CA, USA).

### 2.2. Expression and Purification of HCMV gH/gL and UL128/UL130/UL131A Recombinat Proteins

HCMV strain Merlin sequence (reference # NC_006273.2) was downloaded from NCBI, and gH, gL, UL128, UL130 and UL131A coding sequences were validated and the mutation within UL128 was corrected. For the gH/gL DNA expression construct, the gL sequence encoding amino acid residues (AA) 31-278 and the gH sequence encoding AA 24-718 were linked by a 15 amino acid linker (Gly_4_Ser)_3_ sequence. For the UL128/UL130/UL131A DNA expression construct, the UL128 sequence encoding AA 28-171, UL130 sequence encoding AA 26-214, and UL131A sequence encoding AA 19-129 were linked by (Gly_4_Ser)_3_ sequences in between. An IgG κ leader sequence was added to the 5’ end of gL and UL128 for protein purification, and the sequence encoding His_6_ was added to the 3’ end of gH and UL131A for protein purification. The DNA coding for HCMV gH/gL or UL128/UL130/UL131A proteins were synthesized (Blue Heron Biotech, Bothell, WA, USA) and cloned into pOptiVEC to generate plasmids pOptiVEC-gH/gL or pOptiVEC-UL128/UL130/UL131A and verified by sequencing. CHO cells were transfected with pOptiVEC-gH/gL or pOptiVEC-UL128/UL130/UL131A using Free-style Max reagent and were selected with methotrexate up to the concentration of 4 uM [54,57]. Limiting dilution cloning was used to generate stable clones, and FiberCell bioreactors (FiberCell Systems, Frederick, MD, USA) were used to culture stable CHO cell lines expressing recombinant gH/gL or UL128/UL130/UL131A proteins. Recombinant proteins were purified with affinity chromatography using a cobalt column and size exclusion chromatography using a Superdex 200 column [54,57].

### 2.3. Polyacrylamide Gel Electrophoresis and Western Blot Analysis

Purified HCMV gH/gL and UL128/UL130/UL131A recombinant proteins and cell culture supernatants were analyzed by sodium dodecyl sulfate polyacrylamide gel electrophoresis (SDS-PAGE). Samples were boiled for 10 minutes with 50 mM dithiothreitol, and resolved using SDS running buffer [54,57]. After being transferred to nitrocellulose membranes, the proteins were probed with an anti-gH monoclonal antibody (Santa Cruz Biotechnology, Dallas, TX, USA) or a rabbit anti-UL128 polyclonal antibody [56], followed by goat anti-mouse or goat anti-rabbit secondary antibodies labeled with horseradish peroxidase. After incubation with SuperSignal West Pico chemiluminescent substrate, the signals of the proteins on the nitrocellulose membrane were captured on X-ray film [54,57].

### 2.4. Rabbit Immunizations

Groups of 12–15 week old male New Zealand white rabbits, five in each group, were subcutaneously injected with 25 µg of HCMV recombinant proteins trimeric gB, gH/gL and/or UL128/UL130/UL131A individually or in various combinations (25 µg each). The proteins were mixed with 13 µg aluminum hydroxide and 50 µg CpG-ODN that function as adjuvants [58]. Immunization was performed on day 0, and repeated at week 3 and week 6, and serum samples were taken before initial immunization, 10 days following each immunization and on day 72. These studies were conducted in accordance with the Guide for Care and Use of Laboratory Animals (Institute of Laboratory Animal Resources, NRC, WA, USA), and were approved by the USUHS Institutional Animal Care and Use Committee.

### 2.5. ELISA Analysis of Antigen-Specific Antibodies against HCMV gB, gH/gL and UL128/UL130/UL131A

ELISA plates (Immulon 4) were coated with 5 µg/mL of purified individual HCMV recombinant protein gB, gH/gL or UL128/UL130/UL131A in PBS overnight at 4 °C, followed by blocking with 1% bovine serum albumin (BSA) in PBS. Serum samples were serially diluted in three-fold with 1% BSA-PBS, and were added and incubated in the HCMV protein-coated ELISA plates overnight at 4 °C. The plates were then incubated with a polyclonal goat anti-rabbit IgG labeled with alkaline phosphatase for 1 h at 37 °C, followed by addition of 1 mg/mL alkaline phosphatase substrate (p-nitrophenyl phosphate, disodium) in tris-HCl magnesium-sulfate buffer [54,57]. The plates were washed with 0.1% Tween-20 in PBS between steps, and the absorbance at 450 nm was read on an ELISA reader.

### 2.6. ELISpot Analysis of HCMV Neutralizing Activity

The rabbit immune sera obtained 72 days after immunization with individual or various combinations of HCMV gH/gL, gB, and/or UL128/UL130/UL131A proteins were used for analysis of HCMV neutralizing activity. The rabbit immune sera were either not heat-treated or heat inactivated at 56 ˚C for 30 minutes to eliminate complement activity. An ELISpot assay was used to determine the serum HCMV neutralizing antibody titers as previously described [54,55,57,59,60]. Each serum sample was initially diluted 1:10 and prepared as 1:2 serial dilutions in cell culture medium in triplicates. Each dilution was mixed with an equal volume of culture medium containing 4000 pfu/mL HCMV strains AD169^wt131^, TS15-rN, UxcA66 or clinical isolates 38532 or 39621 [54,55,57], incubated for 4 h at 37 °C. The mixtures of serum and virus were transferred to 96-well plates with a monolayer of ARPE-19 or MRC-5 cells and incubated in 5% CO_2,_ at 37 °C overnight. Following fixation with 200 proof ethanol (≥99.8%), rehydration and blocking with 5% horse serum diluted with PBS, the cells were incubated for 1 h with an anti-IE1 monoclonal antibody, then a secondary goat anti-mouse antibody labeled with biotin for 1 h, and finally incubated for 30 minutes with VECTASTAIN ABC reagent. Between each step, the plates were washed 3x with 0.1% Tween 20 in PBS, and TMB substrate was added in the last step for color development (Mabtech, Inc., Cincinnati, OH, USA). A CTL-ImmunoSpot^®^ S6 Micro Analyzer was used to scan and analyze the plates, and GraphPad Prism7 software was used to calculate the 50% inhibitory concentration (IC_50_). The means of triplicate values for each serum dilution were plotted against log serum dilution, and the best fit four-parameter equation for the data was calculated. The inverse serum dilution interpolated at the mid-point of the curve was the IC_50_ neutralizing titer. For the serum samples that did not have neutralizing activity, neutralizing titer was assigned a value of 1 for the purpose of statistical analyses.

### 2.7. Statistics

For reproducibility, all the experiments were done at least three times. HCMV neutralizing activity titers and the titers of antigen specific antibodies were expressed as geometric means +/− standard error of the mean. Statistical analyses were performed with GraphPad Prism7, p values were determined by two-tailed Students t-test, and *p* < 0.05 was considered significant. 

## 3. Results

### 3.1. Production of HCMV gH/gL and UL128/UL130/UL131A Recombinant Proteins

We previously produced in CHO cells the recombinant HCMV trimeric gB protein, Epstein Barr virus recombinant gB, gH/gL and gp350 proteins [54,57,61,62]. We took a similar approach to produce recombinant HCMV gH/gL and recombinant UL128/UL130/UL131A proteins in the current study. Specifically, a (Gly_4_Ser)_3_ linker coding sequence was inserted between the sequences encoding HCMV gL and gH to let both proteins fold properly, with an IgG ĸ leader coding sequence located at 5’ for the protein to be secreted. Recombinant UL128/UL130/UL131A proteins were expressed similarly, where the coding sequences for UL128, UL130 and UL131A in tandem were separated by a (Gly_4_Ser)_3_ linker coding sequence in between. 

**Figure 1:**
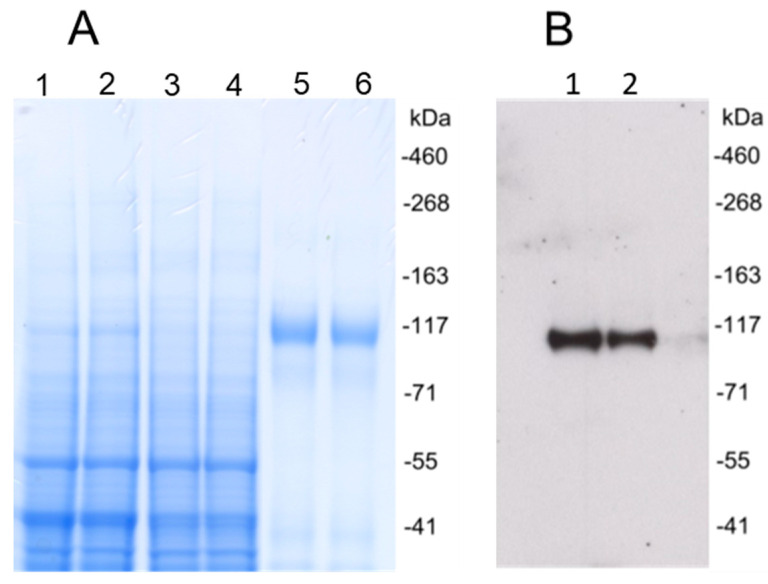
Human cytomegalovirus (HCMV) gH/gL recombinant protein expression and purification. (**A**) Coomassie brilliant blue stained polyacrylamide SDS gel under reducing conditions. Lanes 1 and 2, cell culture supernatant. Lanes 3 and 4, flow-through after Cobalt affinity purification. Lanes 5 and 6, HCMV gH/gL purified by Cobalt affinity purification and size exclusion chromatography. (**B**) Purified HCMV gH/gL recombinant protein was analyzed with Western blot under reducing conditions using an anti-gH monoclonal antibody. Lanes 1 and 2, purified proteins.

**Figure 2:**
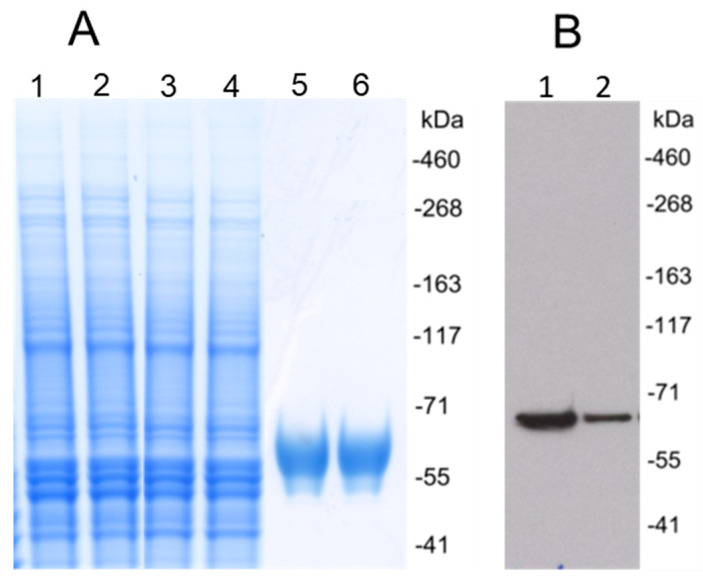
Expression and purification of HCMV UL128/UL130/UL131A recombinant protein. (**A**) Coomassie brilliant blue stained polyacrylamide SDS gel under reducing conditions. Lanes 1 and 2, cell culture supernatant. Lanes 3 and 4, flow-through after Cobalt affinity purification. Lanes 5 and 6, UL128/UL130/UL131A recombinant protein purified using Cobalt affinity purification and followed by size exclusion chromatography. (**B**) Western blot analysis of HCMV UL128/UL130/UL131A recombinant protein using anti-UL128 polyclonal antibodies under reducing conditions. Lanes 1 and 2, purified proteins.

Synthesized DNA coding for recombinant HCMV gH/gL or UL128/UL130/UL131A protein was cloned into pOptiVEC vector and transfected CHO cells. Stable CHO cell lines expressing gH/gL or UL128/UL130/UL131A were generated by limiting dilution cloning. Recombinant proteins were purified from supernatants of CHO cell cultures using affinity and size exclusion chromatography. Purified gH/gL protein was analyzed by Western blot under reducing conditions using a monoclonal anti-HCMV gH antibody, and showed a single size ~110 kDa band, consistent with the predicted size of the heterodimeric gH/gL (Figure 1). Western blot analysis of the UL128/UL130/UL131A protein showed a band of ~57 kDa under reducing conditions, which was consistent with the predicted size of UL128/UL130/UL131A (Figure 2). 

### 3.2. Immunization of Rabbits with HCMV Trimeric gB, gH/gL or UL128/UL130/UL131A Recombinant Proteins Each Induced High Serum Titers of Antigen-specific IgG, with No Interference in the Induction of Individual Antigen-specific IgG Following Immunization with Protein Combinations 

**Figure 3:**
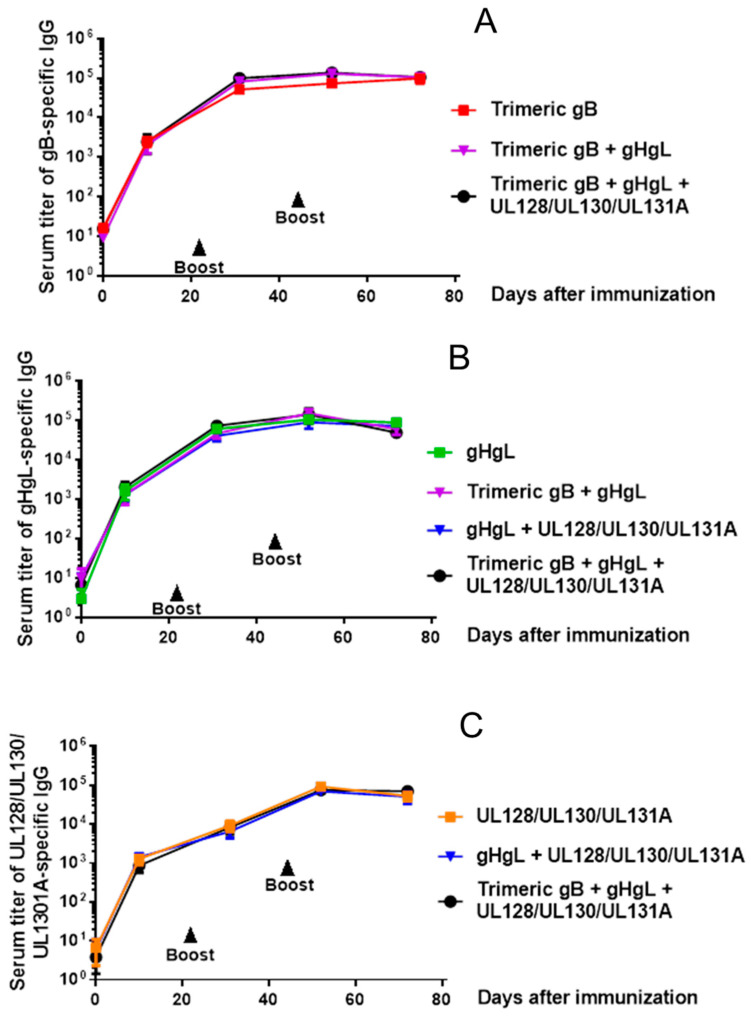
Immunization of rabbits with HCMV trimeric gB, gH/gL and/or UL128/UL130/UL131A recombinant proteins induced high serum titers of antigen-specific IgG, without interference when used in combination. Groups of 12–15 week old rabbits (*n* = 5), were subcutaneously immunized with 25 µg of recombinant trimeric HCMV gB, HCMV gH/gL or UL128/UL130/UL131A individually or in various combinations (25 µg each) adjuvanted with alum + CpG-ODN, then boosted on days 21 and 42. Serum samples were obtained 10 days following each immunization and on day 72 for measurement of serum titers of antigen-specific IgG by ELISA. (**A**) Serum titers of gB-specific IgG. (**B**) Serum titers of gH/gL-specific IgG. (**C**) Serum titers of UL128/UL130/UL131A-specific IgG.

Groups of adult rabbits, five in each group, were subcutaneously immunized with 25 µg of HCMV trimeric gB, gH/gL or UL128/UL130/UL131A recombinant protein individually or in various combinations of these recombinant proteins (25 µg each) using alum + CpG-ODN as adjuvants. Rabbits were then boosted on days 21 and 42 in a similar fashion. As shown in Figure 3A–C, immunization of rabbits with trimeric HCMV gB, HCMV gH/gL or UL128/UL130/UL131A individually induced high serum IgG titers (~1:100,000) of antigen-specific antibodies. The titers of anti-gB IgG and anti-gH/gL IgG induced by immunization with trimeric gB in combination with gH/gL were not significantly different from the IgG titers induced by immunization with trimeric gB or gH/gL alone (Figure 3A,B). The titers of anti-gH/gL IgG and anti-UL128/UL130/UL131A IgG induced by immunization with the combination of gH/gL and UL128/UL130/UL131A were not significantly different from the IgG titers induced by immunization with gH/gL or UL128/UL130/UL131A alone (Figure 3A,B). Using the trivalent combination of trimeric gB, gH/gL and UL128/UL130/UL131A for immunization induced high titers of antigen-specific IgG against gB, gH/gL and UL128/UL130/UL131A. The titers of anti-gB IgG, anti-gH/gL IgG or anti-UL128/UL130/UL131A IgG were not significantly different from that induced by immunization with corresponding proteins individually (Figure 3A–C). 

### 3.3. Immunization of Rabbits with HCMV gB in Combination with gH/gL Demonstrated Strong Synergistic Effects in Elicitation of Neutralizing Activity for Prevention of HCMV Infection of Fibroblasts, Whereas Addition of UL128/UL130/UL131A Had No Effect

**Figure 4:**
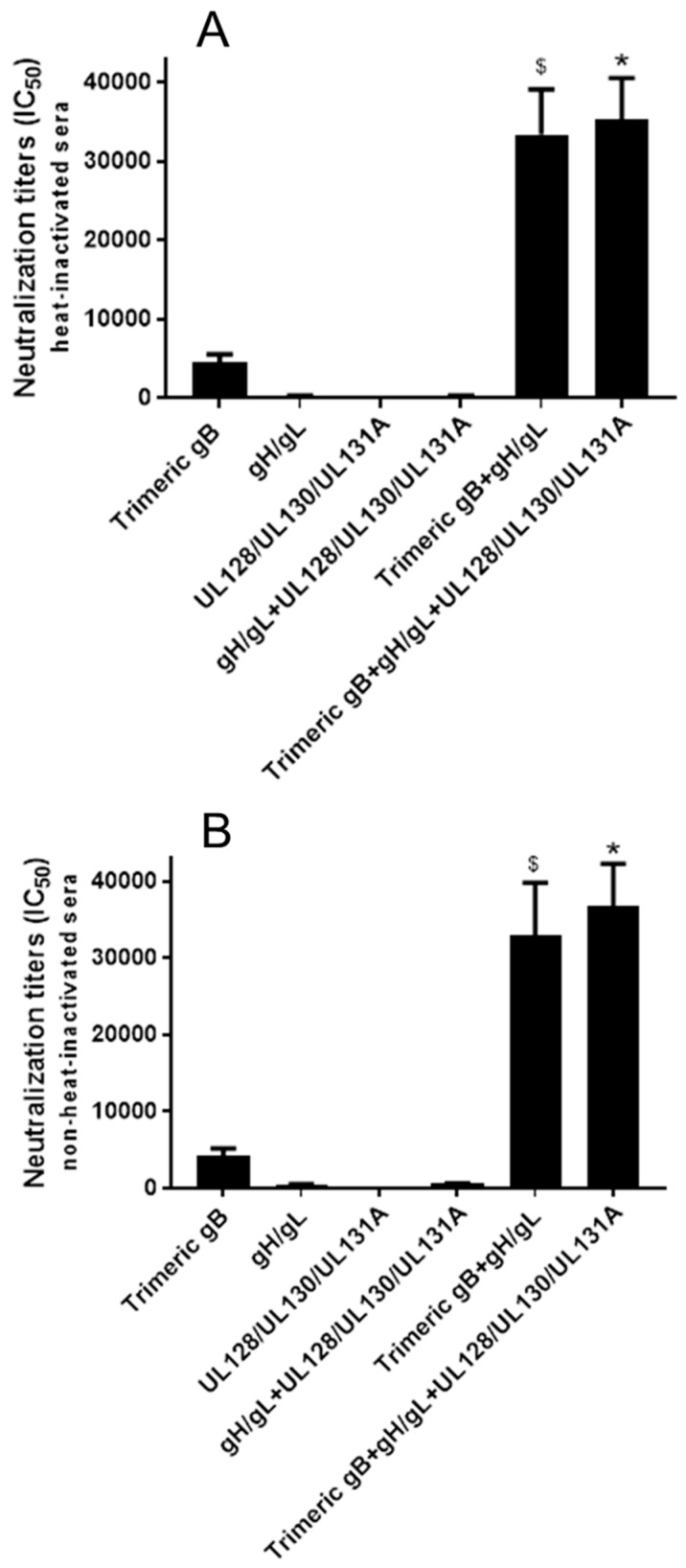
Immunization of rabbits with HCMV gB in combination with gH/gL elicited strong synergistic neutralizing activity preventing HCMV infection of fibroblasts. Day 72 immune sera from rabbits (*n* = 5) subcutaneously immunized three times with 25 µg of recombinant trimeric HCMV gB, HCMV gH/gL and UL128/UL130/UL131A individually or in various combinations (25 µg each) adjuvanted with alum + CpG-ODN were either heat-inactivated (**A**) or non-heat-inactivated (**B**). IC_50_ neutralizing activities were determined using MRC-5 fibroblasts and HCMV strain AD169^wt131^. Significance $, * *p* < 0.05 compared to the sum of HCMV neutralizing activities of the sera from rabbits immunized with individual envelope proteins.

The HCMV neutralizing activities of day 72 sera from rabbits immunized with three doses of 25 µg of HCMV trimeric gB, gH/gL, UL128/UL130/UL131A individually or in various combinations (25 µg each) were first analyzed using MRC-5 fibroblasts and HCMV strain AD169 with the UL131A gene repaired (AD169^wt131^). The initial analyses were conducted on sera subjected to heat-inactivation at 56 °C for 30 minutes to eliminate complement activity (Figure 4A). The pre-immune rabbit sera and the sera from rabbits injected with adjuvants alone did not show detectable HCMV neutralizing activity. Trimeric gB alone elicited high titer of HCMV neutralizing activity (IC_50_ 4511), gH/gL alone elicited moderate neutralizing activity (IC_50_ 257), and UL128/UL130/UL131A alone elicited very low neutralizing activity (IC_50_ 2) for MRC-5 fibroblasts (Figure 4A). Immunization of rabbits with HCMV trimeric gB in combination with gH/gL elicited ~8-fold higher neutralizing activity than the sum of the neutralizing activities elicited by trimeric gB and gH/gL individually. In contrast, immunization with gH/gL in combination with UL128/UL130/UL131A did not elicit higher neutralizing activity compared to the neutralizing activity elicited by gH/gL alone. Similarly, immunization of rabbits using the trivalent combination of trimeric gB, gH/gL and UL128/UL130/UL131A did not elicit higher neutralizing activity than that of immunization with trimeric gB in combination with gH/gL, consistent with UL128/UL130/UL131A not being required for HCMV infection of fibroblasts (Figure 4A). Analysis of immune sera without heat-inactivation to preserve complement activity showed slightly increased HCMV neutralizing activities, but otherwise gave similar results in regard to the synergistic effects of the gB + gH/gL combination (Figure 4B).

### 3.4. Immunization with Combinations of HCMV Recombinant Proteins gB, gH/gL and UL128/UL130/UL131A Showed Strong Synergistic Effects in Elicitation of Neutralizing Activity for Prevention of HCMV Infection of Epithelial Cells

**Figure 5:**
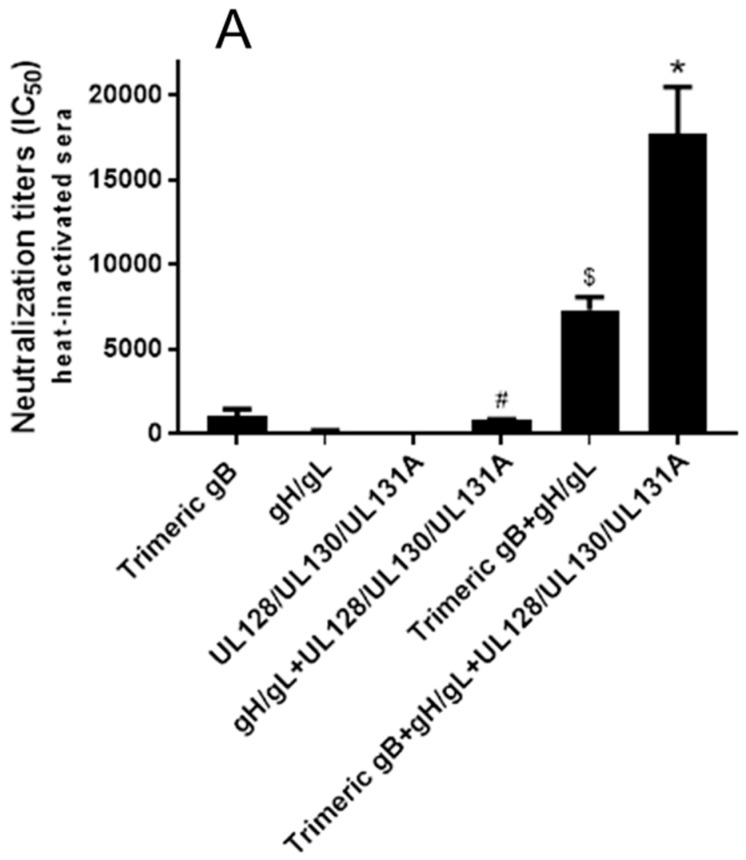

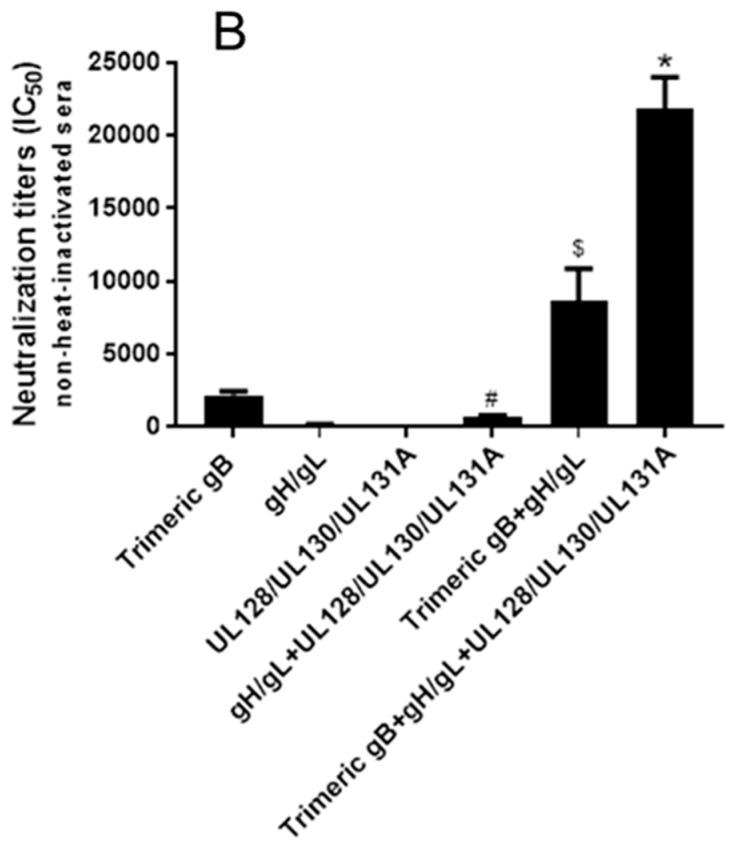
Immunization with combinations of HCMV gB, HCMV gH/gL and/or UL128/UL130/UL131A proteins induced strong synergistic neutralizing activities preventing HCMV infection of epithelial cells. Day 72 immune sera from rabbits (*n* = 5) subcutaneously immunized three times with 25 µg of trimeric HCMV gB, HCMV gH/gL and UL128/UL130/UL131A individually or in various combinations (25 µg each) adjuvanted with alum + CpG-ODN were either heat-inactivated (**A**) or non-heated (**B**). IC_50_ neutralizing activities were determined using HCMV strain AD169^wt131^ and the ARPE-19 epithelial cell line. Significance #, $, * *p* < 0.05 compared to the sum of HCMV neutralizing activities of the sera from rabbits immunized with individual envelope proteins.

Using HCMV strain AD169^wt131^ and ARPE-19 epithelial cells, the day 72 rabbit immune sera obtained after immunization with three doses of 25 µg of trimeric HCMV gB, HCMV gH/gL and UL128/UL130/UL131A individually or in various combinations (25 µg each) were further analyzed for HCMV neutralizing activities. Similar to the results obtained with MRC-5 fibroblasts, using heat-inactivated sera, the sera from rabbits immunized with trimeric gB alone demonstrated high titers of HCMV neutralization (IC_50_ 1067), immunization with gH/gL alone elicit moderate neutralizing activity (IC_50_ 179) and immunization with UL128/UL130/UL131A alone elicited very low neutralizing activity (IC_50_ 2) (Figure 5A). The pre-immune rabbit sera and the sera from rabbits injected with adjuvants alone did not show detectable serum HCMV neutralizing activity. Immunization with the combination of gH/gL and UL128/UL130/UL131A elicited ~4-fold higher neutralizing activity than the sum of the neutralizing activities elicited by gH/gL and UL128/UL130/UL131A individually (Figure 5A). Immunization with trimeric gB in combination with gH/gL induced ~6-fold higher neutralizing activity than the sum of the neutralizing activities elicited by gB and gH/gL individually (Figure 5A). Immunization of rabbits using the trivalent combination of trimeric gB, gH/gL and UL128/UL130/UL131A elicited ~18-fold higher neutralizing activity than the sum of the neutralizing activities elicited by gB, gH/gL and UL128/UL130/UL131A individually, an additional ~3-fold increase compared to that elicited by immunization with trimeric gB in combination with gH/gL (Figure 5A). Without heat-inactivation in order to preserve complement activity, a modest increase in serum HCMV neutralizing activities was observed, with similar synergistic effects in induction of serum HCMV neutralizing activity following immunization with the combinations of HCMV envelope proteins (Figure 5B).

### 3.5. Immunization with Combinations of HCMV gB, HCMV gH/gL and/or UL128/UL130/UL131A Recombinant Proteins Induced Strong Synergistic Neutralizing Activity Preventing HCMV Clinical Isolates from Infection of Fibroblasts and Epithelial Cells

**Figure 6:**
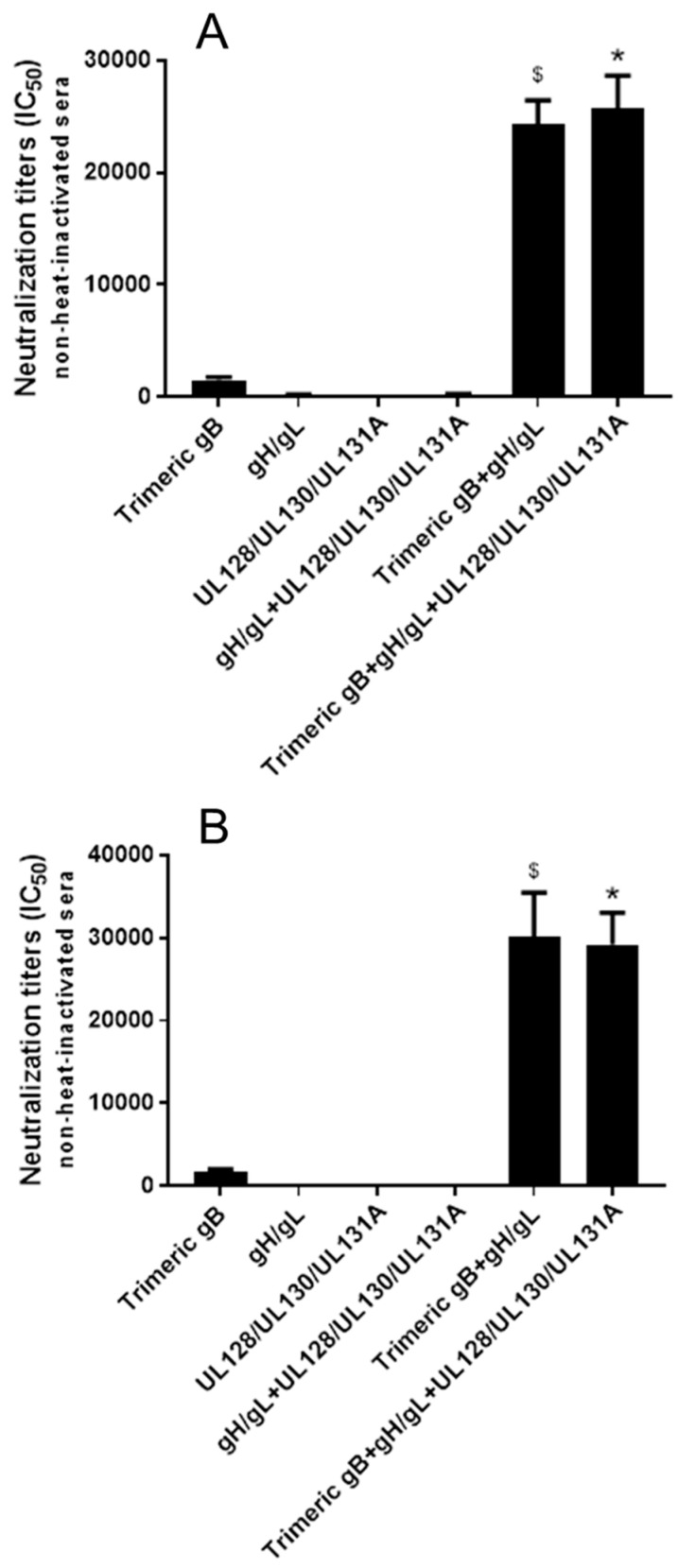

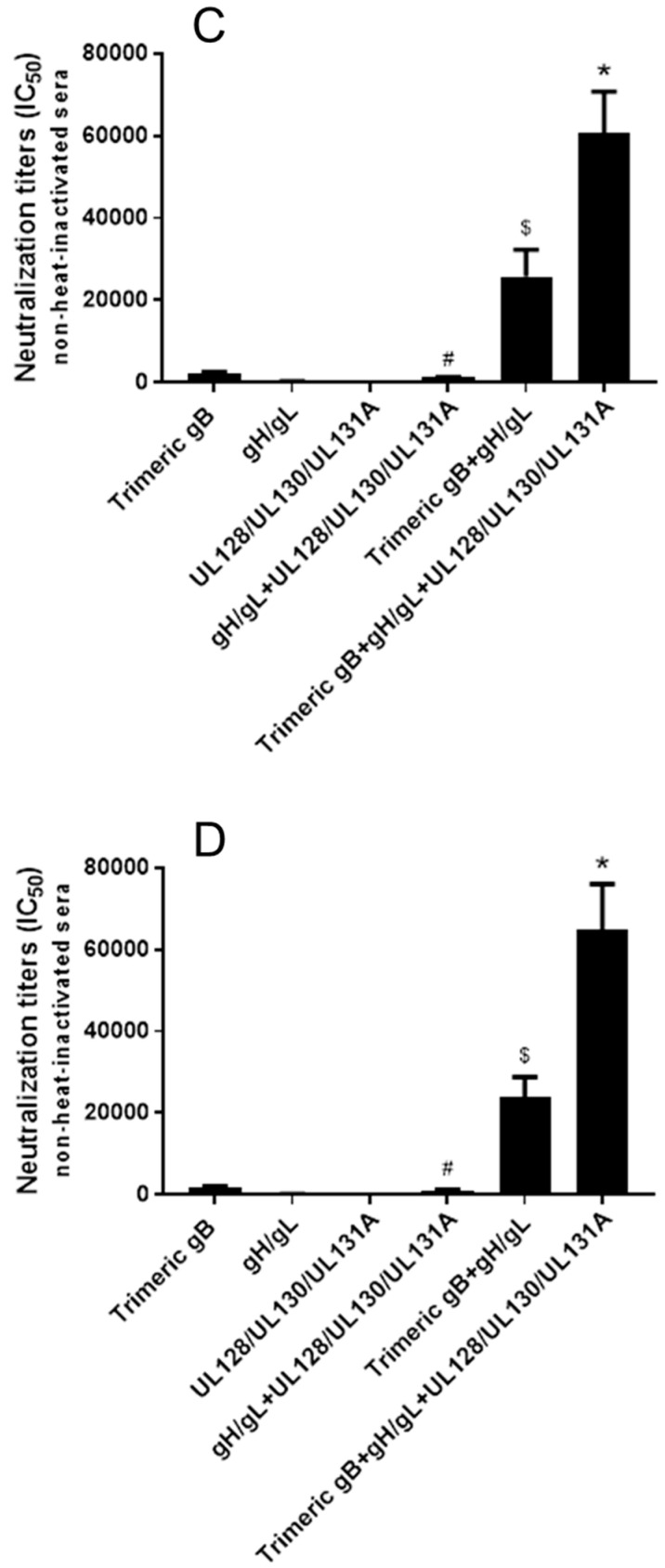
Immunization of rabbits with HCMV gB in combination with gH/gL elicited strong synergistic neutralizing activity against HCMV clinical isolates propagated in fibroblasts, whereas using the trivalent combination of trimeric gB, gH/gL and UL128/UL130/UL131A for immunization demonstrated strong synergistic neutralizing activity against HCMV strains propagated in epithelial cells. Day 72 immune sera from rabbits (*n* = 5) subcutaneously immunized three times with 25 µg of recombinant trimeric HCMV gB, HCMV gH/gL, UL128/UL130/UL131A individually or in various combinations (25 µg each) adjuvanted with alum + CpG-ODN were non-heat-inactivated. IC_50_ neutralizing activities were determined using MRC-5 fibroblasts and fibroblast adapted HCMV clinical isolates 38532 (**A**) and 39621 (**B**), or the ARPE-19 epithelial cells and epithelial cell adapted HCMV strains TS15-rN (**C**) and UxcA66 (**D**). Significance #, $, * *p* < 0.05 compared to the sum of HCMV neutralizing activities of the sera from rabbits immunized with individual HCMV envelope proteins.

HCMV neutralizing activities of the rabbit sera after immunization with HCMV recombinant trimeric gB, gH/gL, and UL128/UL130/UL131A individually or in various combinations (25 µg each) were determined using HCMV clinical isolates propagated in MRC-5 fibroblasts or HCMV strains propagated in ARPE-19 epithelial cells. As heat inactivation did not show significant effects on the neutralizing activities of rabbit immune sera when analyzed with HCMV strain AD169^wt131^, the neutralizing activities against HCMV clinical isolates and additional HCMV strains were analyzed using day 72 rabbit immune sera without heat inactivation. Similar to the results obtained with HCMV strain AD169^wt131^, using HCMV clinical isolates 38532 and 39621 propagated in MRC-5 fibroblasts, immunization with trimeric gB alone elicited high titer HCMV neutralizing activity (IC_50_ 1508 and 1717), immunization with gH/gL alone elicited moderate neutralizing activity (IC_50_ 224 and 187), and immunization with UL128/UL130/UL131A alone elicited very low neutralizing activity for MRC-5 fibroblasts (IC_50_ 2 and 3) (Figure 6A,B). Compared to the sum of the neutralizing activities elicited by gB and gH/gL individually, immunization with gB in combination with gH/gL elicited ~13-fold and ~17-fold higher neutralizing activities against HCMV clinical isolates 38532 and 39621, respectively. Similar to the results obtained using AD169^wt131^, adding UL128/UL130/UL131A to either the gB + gH/gL combination or gH/gL alone elicited no additional serum neutralizing activity for prevention of HCMV clinical isolates 38532 or 39621 from infection of fibroblasts (Figure 6A,B).

The HCMV neutralizing activities of day 72 sera from rabbits immunized with three doses of 25 µg of HCMV trimeric gB, gH/gL, and UL128/UL130/UL131A individually or in various combinations (25 µg each) were further analyzed using HCMV strains TS15-rN and UxcA66 and ARPE-19 epithelial cells. Without heat inactivation, the sera from rabbits immunized with trimeric gB alone demonstrated high titer HCMV neutralizing activity (IC_50_ 2399 and 1804), rabbit sera obtained from immunization with gH/gL alone showed moderate HCMV neutralizing activity (IC_50_ 247 and 211) and sera from rabbits immunized with UL128/UL130/UL131A alone had low neutralizing activity (IC_50_ 6 and 11) (Figure 6C,D). Immunization with the combination of gH/gL and UL128/UL130/UL131A elicited ~5-fold higher neutralizing activity than the sum of the neutralizing activities elicited by gH/gL and UL128/UL130/UL131A individually for epithelial cells (Figure 6C,D). Compared to the sum of the neutralizing activities elicited by gB and gH/gL individually, immunization with gB in combination with gH/gL elicited ~11- and ~13-fold higher neutralizing activities for HCMV strains TS15-rN and UxcA66, respectively (Figure 6C,D). Using the trivalent combination of trimeric gB, gH/gL and UL128/UL130/UL131A for immunization elicited ~25 and ~32-fold higher neutralizing activities against HCMV strains TS15-rN and UxcA66 respectively than the sum of the neutralizing activities elicited by gB, gH/gL and UL128/UL130/UL131A individually (Figure 6C,D). 

## 4. Discussion

HCMV requires multiple envelope proteins to bind to and enter host cells via fusion of the viral envelope with the cell endosomal or plasma membrane. HCMV envelope glycoproteins gB, gH, gL, gO and a group of small accessory proteins UL128, UL130, UL131A have been shown to play key roles in HCMV infection of host cells [20,22]. The HCMV gB protein is identified as the direct mediator of viral envelope fusion with host cell endosomal or plasma membrane, and the association of the gH/gL/gO protein complex with gB is required for activation of its fusogenic activity, where PDGFα on the surface of host cells serves as the receptor for gH/gL/gO complex [24,25,26,29,30]. For efficient targeting of HCMV to epithelial and endothelial cells, the pentameric complex gH/gL/UL128/UL130/UL131A is required, which mediates HCMV binding to cell surface receptors [20,23,31,32]. The pentameric complex has also been shown to play an important role in cell–cell transmission of HCMV, which is resistant to neutralizing antibodies [63]. It is still controversial whether the gH/gL/gO complex plays a role in HCMV infection of epithelial cells, as the expression of PDGFα on the epithelial cell surface is very low [30]. However, the HCMV conserved core fusion machinery, consisting of envelope glycoproteins gB, gH and gL, are believed to be required for HCMV entry and infection of all its target cells. 

The third HCMV envelope protein complex consisting of gH/gL—the gB/gH/gL complex—has been reported, following the discovery of the pentameric complex and the gH/gL/gO complex [64]. Vanarsdall et al. reported that in the endoplasmic reticulum shortly after their synthesis, gB bound to gH/gL to form the gB/gH/gL complex. In the envelope of HCMV virions, up to 50% gH/gL was found to bind to gB, which makes the gB/gH/gL protein complex the dominant gH/gL protein complex of the three [64]. It has been hypothesized that the gB in the gB/gH/gL complex could be stabilized in pre-fusion confirmation, but the function of the gB/gH/gL complex is currently not clear [30,64]. It was previously believed that the gB/gH/gL complex only existed transiently during HCMV envelope fusion with host cell membrane, but this study provided critical evidence that gB/gH/gL exists as a stable protein complex in the envelope of HCMV virions [64]. As the HCMV core fusion machinery envelope proteins gB, gH and gL are all required for HCMV to enter and infect all the target cells, the identification of the gB/gH/gL complex in the envelope of HCMV virions makes it a unique HCMV vaccine candidate. Just like the pentameric complex, the gB/gH/gL complex may induce neutralizing antibodies against the conformational epitopes formed by all the three core fusion machinery proteins and provide highly efficient broad protection against HCMV infection. 

HCMV gB and the pentameric complex are extensively studied as vaccine candidates due to their critical role in HCMV entry into and infection of target cells. The first phase II placebo-controlled study of a gB/MF59 vaccine in postpartum HCMV-seronegative women demonstrated 50% efficacy against primary HCMV infection [19]. The following second phase II multi-center study with the gB/MF59 vaccine in healthy HCMV-seronegative adolescent girls showed 43% efficacy in preventing primary HCMV infection [42]. In a third phase II study conducted in solid-organ transplant recipients, when compared to a placebo control the gB/MF59 vaccine reduced viremia as well as the number of days requiring treatment with ganciclovir [43]. Further, in HCMV-seronegative patients who received HCMV-positive transplants, the viremia was inversely correlated with the gB-specific antibody [43]. Several clinical trials and pre-clinical studies have evaluated HCMV candidate vaccines incorporating the pentameric complex. A replication defective live attenuated HCMV vaccine expressing the pentameric complex elicited higher HCMV neutralizing titers than that of naturally HCMV seropositive subjects in a phase I clinical trial [34,65]. Immunization of mice with an MVA vector expressing the HCMV pentameric complex induced potent HCMV neutralizing activities that were both complement-dependent and complement-independent [66]. Also, in mice and nonhuman primates, immunization with HCMV mRNAs encapsulated with lipid nanoparticles that encode the five components of the pentameric complex induced long-lasting high titers of HCMV neutralizing activity [67]. 

The phase II clinical trials with HCMV gB/MF59 vaccine demonstrated ~50% efficacy in prevention of HCMV infection in both immunocompetent and immunocompromised subjects and represent a milestone in vaccine development against HCMV [34,35,36]. However, the recombinant HCMV gB protein (Chiron gB) used in these clinical trials does not recapitulate the native HCMV gB trimeric conformation. Thus, generation of a HCMV trimeric gB that expresses native conformational epitopes could significantly improve the immunogenicity of recombinant HCMV gB. We previously produced a HCMV recombinant trimeric gB that induced 11-fold higher gB-specific IgG serum titers and 50-fold higher titers of cross-strain HCMV neutralizing activities compared to a recombinant gB protein similar to the HCMV gB used in the above clinical trials [54]. In the current study, we demonstrated that immunization of rabbits with 25 µg of trimeric HCMV gB elicited high titers of HCMV neutralizing activity not only for MRC-5 fibroblasts but also for ARPE-19 epithelial cells. In contrast, in our prior mouse studies immunization with the same amount of HCMV gB only elicited high titers of HCMV neutralizing activity for MRC-5 fibroblasts but moderate titers of HCMV neutralizing activity for ARPE-19 epithelial cells, a result most likely caused by species differences [54,56]. Heat inactivation did not show significant effects on the HCMV neutralizing activity of the rabbit immune sera analyzed with HCMV strain AD169^wt131^_._ Therefore, subsequent HCMV neutralizing analysis with HCMV clinical isolates 38532 and 39621 and HCMV strains TS15-rN and UxcA66 were performed using rabbit immune sera without heat inactivation. 

Though the pentameric complex elicits a high titer of HCMV neutralization activity, the antibodies elicited are protective against HCMV infection of epithelial cells, endothelial cells and monocytes, but not fibroblasts or primary trophoblast progenitor cells [46,47,48,49,68,69,70,71]. It has been suggested that the combination of trimeric gB and pentameric complex proteins may be an optimal prophylactic HCMV vaccine [37,72,73]. The effect of gB in combination with pentameric complex has been evaluated using MVA vectored or RNA vaccine that simultaneously express gB and the pentameric complex [66,67]. Although enhanced HCMV neutralizing activities were elicited in these studies, neutralizing activities induced by the pentameric complex were dominant, and no synergistic or additive effect was observed [66,67]. In the current study, we are the first to report that immunization with gB in combination with gH/gL can induce strong synergistic neutralizing activities against HCMV infection of both fibroblasts and epithelial cells. Specifically, immunization with recombinant HCMV trimeric gB in combination with gH/gL elicited ~8-, ~13 and ~17-fold higher HCMV neutralizing activities for fibroblasts against HCMV AD169^wt131^ and HCMV clinical isolates 38532 and 39621, respectively, as compared to the sum of neutralizing activities elicited by HCMV trimeric gB or gH/gL alone. For epithelial cells, immunization with recombinant HCMV trimeric gB in combination with gH/gL elicited ~6-, ~11 and ~13-fold higher HCMV neutralizing activities for HCMV AD169^wt131^, TS15-rN and UxcA66, respectively, compared to the sum of neutralizing activity elicited by HCMV trimeric gB or gH/gL individually. In light of the study by Vanarsdall et al.—which showed that gB/gH/gL is the dominant gH/gL protein complex in the HCMV virion envelope, whereas gB, gH and gL consist of the core fusion machinery and are required for HCMV infection of all its target cells—the gB/gH/gL complex is a promising HCMV vaccine candidate [64]. However, production of the gB/gH/gL complex may be technically difficult. Production of gB and gH/gL separately and using gB in combination with gH/gL as a vaccine would be an attractive alternative. Whether mixing of gB and gH/gL leads to complex formation remains to be determined. 

We also demonstrated that even stronger synergistic HCMV neutralizing activities were induced by the combination of trimeric gB, gH/gL and UL128/UL130/UL131A for preventing HCMV infection of epithelial cells. Using the trivalent combination of trimeric gB, gH/gL and UL128/UL130/UL131A for immunization resulted in about 18-, 25- and 32-fold higher HCMV neutralizing activities respectively for HCMV AD169^wt131^, TS15-rN and UxcA66, compared to the sum of neutralizing activities elicited by individual proteins. These data suggest that the combination of HCMV trimeric gB and the pentameric complex would likely be an optimal vaccine against HCMV infection. Our finding that strong synergistic HCMV neutralizing activity can be elicited by the combination of HCMV gB, gH/gL, the core fusion machinery envelope proteins, and/or accessory proteins UL128/UL130/UL131A is critical for future HCMV vaccine design. Immunization with a combination of these HCMV envelope proteins not only provides broader protection to cover potentially all the HCMV target cells, it also markedly enhances the HCMV neutralizing activities elicited compared to individual envelope proteins or protein complexes.

Immunization with individual envelope protein trimeric HCMV gB, HCMV gH/gL or UL128/UL130/UL131A each induced high serum titers of antigen-specific IgG, and immunization with various combinations of these proteins did not lead to interference in the induction of individual antigen-specific IgG responses, consistent with the findings of other investigators [56,66]. Non-neutralizing antibody functions may also play important roles during virus infection, and antibody-dependent cell-mediated phagocytosis has been implicated in prevention of HCMV infections [66,74,75]. In future studies, in addition to their potent neutralizing activity, the non-neutralizing antibody functions of the high titer antibodies induced by immunization with the combination of HCMV trimeric gB, gH/gL, the core fusion machinery envelope proteins, and/or accessory proteins UL128/UL130/UL131A should be assessed [66,74,75].

## 5. Conclusions

Significant morbidity and mortality are caused by congenital HCMV infection and HCMV infection of immunosuppressed patients, and development of an HCMV vaccine is a major public health priority. Natural immunity against HCMV infection is only partially protective. Subunit vaccines based on purified recombinant HCMV proteins can elicit qualitatively or quantitatively different immune responses from those induced by natural HCMV infection. Immunization with recombinant HCMV core fusion machinery envelope proteins trimeric gB in combination with gH/gL or the combination of trimeric gB + the pentameric complex could induce strong synergistic HCMV neutralizing activity. This will be an efficient and safe approach for the development of an HCMV vaccine that could potentially provide superior protection than natural immunity acquired from HCMV infection.
